# When Is an Interview an Inter View? The Historical and Recent Development of Methodologies Used to Investigate Children’s Astronomy Knowledge

**DOI:** 10.1007/s11165-021-10032-8

**Published:** 2022-01-01

**Authors:** Eric J. Blown, Tom G. K. Bryce

**Affiliations:** University of Strathclyde, Glasgow, Scotland, UK

**Keywords:** Historical review, Interview methodology, Children’s cosmologies, Cognitive development, Dynamism of memory, Socratic dialogue

## Abstract

This paper provides a historical review of the interview research that has been used by science educators to investigate children’s basic astronomy knowledge. A wide range of strategies have been developed over the last 120 years or so as successive teams of researchers have endeavoured to overcome the methodological difficulties that have arisen. Hence, it looks critically at the techniques that have been developed to tackle the problems associated with interviews, questionnaires and tests used to research cognitive development and knowledge acquisition. We examine those methodologies which seem to yield surer indications of how young people (at different ages) understand everyday astronomical phenomena—the field often referred to as *children*’*s cosmologies*. Theoretical ideas from cognitive psychology, educational instruction and neuroscience are examined in depth and utilised to critique matters such as the importance of subject mastery and pedagogical content knowledge on the part of interviewers; the merits of multi-media techniques; the roles of open-ended vs. structured methods of interviewing; and the need always to recognise the dynamism of memory in interviewees. With illustrations and protocol excerpts drawn from recent studies, the paper points to what researchers might usefully tackle in the years ahead and the pitfalls to be avoided.

## When Is an Interview an *Inter View*?

Kvale and Brinkmann (2014) open their insightful guide to interview research (a well-researched text in itself, now in its 3^rd^ Edition) with the statement:

If you want to know how people understand their world and their lives, why not talk to them? Conversation is a basic mode of human interaction. Human beings talk with each other; they interact, pose questions, and answer questions … (p. xvii).

They stress that an interview is an *inter view*, where ‘knowledge is constructed in the inter-action between the interviewer *and* the interviewee’ (p. viii), an argument which we agree with and seek to develop in several ways in this paper. Historically, interview methodology has progressed significantly, especially in investigations into children’s intellectual development—thus the work of Piaget and Vygotsky through to Donaldson, Bruner and beyond. A particularly fruitful avenue of this research has been that associated with young people’s cosmological ideas which have been the focus of many researchers; concepts like daytime and night-time, sunrise and sunset, the movement of the Earth, Sun and Moon (ESM), seasons and so forth—pioneered by Piaget and continued by others, including Nussbaum and Novak et al., Vosniadou and Brewer et al., Nobes et al., Schoultz, Säljö et al. and the current authors. An important feature of this development has been the early trend away from *replicable* standardized tests (Binet et al.), through *replicable* clinical interviews (Piaget et al.) originally aimed at supporting ‘ages and stages’, to guided, semi-structured, open-ended interviews with Socratic dialogue; the latter by their very nature being *non-replicable* (see further below). This evolution is countered by a more recent trend utilising cultural artefacts such as globes, maps and pre-made models of the Earth in interviews featuring forced-choice rather than open-ended questions. It is apparent from the literature examined below that the interview has evolved from objective, rather sterile, non-interactive origins requiring minimal subject/content knowledge (CK), or pedagogical content knowledge (PCK), on the part of the researcher, to a more subjective, constructive and interactive activity requiring maximal CK and PCK on the part of the interviewer.

We believe that the review presented here is timely because researchers are now at the stage of being able to correlate neuroscience with psychology in response to interview questions. Preliminary findings indicate that memory (*what* young people seem to recall) is much more dynamic and creative than previously thought and sensitive, open-ended questioning is an essential tool in probing this dynamism. Thus we seek to encourage debate on changing views of cognitive development in general and memory in particular as a result of these recent advances in neuroscience, developments which challenge the reproductive aspects of memory (traditionally reflected in evidence from Piagetian interviews with Socratic dialogue). In Bryce & Blown (2016), we gave a first account of how ideas are dynamic and multimodal, actively created at the point of recall (*cf.* Edelman, 2001), demonstrating the extent to which children switched between what they said, what they drew and what they modelled in play-dough during interviews. And in Bryce & Blown (2020), we illustrated the conceptual flexibility apparent in children’s interview responses, showing the creation as well as the *inhibition* of concepts (see footnote^1^ and further in due course).

The studies of *children*’*s cosmologies* and the frameworks used here were selected because they utilised Piagetian open-ended, or socio-cultural forced-choice interviews, to investigate children’s developing concepts, the considered nature of which, in fact, reflect *methodological* issues. Some of the literature scrutinised below is in opposition to the authors’ arguments and an effort has been made to maintain a balance across the review.

In our analysis, the overarching question has been to identify what can now be claimed as productive features of in-depth interviewing and, *inter alia*, the characteristics of effective interviewers—the knowledge and skills required of them, whether as researchers or teachers, roles which often overlap in efforts to ascertain children’s intuitive and scientific ideas. We will therefore focus critically on:

- (a) The need for interviewers to be involved in any research design so that they are better prepared for opportunities to diverge from standard interview questions to explore concepts more deeply using Socratic dialogue;
- (b) The significant part played by *multimodal* interview sessions and what can be interpreted from interviewees’ responses to questioning utilising *different* modalities;
- (c) How an understanding of the creative (as opposed to the reproductive) dimension of remembering—what recent neuroscience research tells us about the *dynamism* of human memory—alters our interpretations of what may be revealed when people are questioned (see footnote ^1^);
- (d) The consideration which should be given to the value of the spoken word as a reflection of conceptual ideas and the merits of open-ended over forced-choice questions.

On the interviewer as teacher or teacher as interviewer raised in (a), sensitive questioning requires robust CK/PCK and must be carried out carefully to avoid ‘foreclosure’ on judgements about subjects’ understandings. Allowing for the creative aspects of remembering, interviewers should always assume that an interviewee probably has more to say/to mean and the interview must open up the prospects for him/her doing so (*c.f.* the exemplifications discussed in Bryce & Blown, 2021). Through a close examination of how (a) and (b) have figured in much of the science education literature—particularly in studies of children’s cosmologies—together with the as-yet-few acknowledgments of (c) (dynamic memory) in most interview research, we will argue that researchers (and teachers) can misrepresent or pre-judge the understandings which young people have of basic concepts. Our discussion will *juxtapose* Socratic questioning and the PCK of the *interviewer* with the creative dimension to remembering in the *interviewee*. With respect to (d), although we place high value on the spoken word as a reflection of thought, we are aware of its limitations particularly with younger children and those of other cultures. Because of this in our own cross-age, cross-cultural work we used Socratic dialogue to clarify verbal responses to questions and triangulated verbal language with children’s drawings and play-dough models, as detailed below.

## A Historical Review of Investigations into Children’s Cosmologies

The following is a brief outline of the many studies which have been conducted in the last 120 + years, listed chronologically, where children have been interviewed in attempts to reveal how they understand natural phenomena.^2^ In the short discussion of each investigation, we discuss the key methodology employed and, where appropriate, compare and contrast the reasons indicated by the researchers concerned—being respectful and sensitive to the very different circumstances in which the early researchers operated. Contrasts become particularly evident in the studies carried out in the last two decades (e.g. between Vosniadou et al., and ourselves vs. Schoultz et al.) and we make these clear.

Beginning just before the last century, Lange (1879) pioneered the use of questionnaires and interviews to investigate 800 young children’s ideas about the natural world on entering school in Plauen, Germany. The studies seemed to indicate how little children knew about things and how much needed to be done in schools. The researchers found that kindergarten children knew a little more than non-kindergarten children, and there were some sex differences. City and country children were also found to be different. For example, Lange reported that 42% of country children had seen the Sun rise vs. only 18% of city children, and that 70% of country children had reported seeing and hearing a lark *vs*. only 20% of city children (see in Reese, 1982).

A few years later, Hall (1883) conducted the first cross-cultural studies of children’s cosmologies with 400 Anglo-American, Irish-American and local German children, aged 3–6 years in Boston, USA. Reese (1982) observes that Hall seemed to have been quite sensitive to questions of method and took much more care than the antecedent Germans had. Noting the rate of the German work, about one question every half-minute, Hall had suggested that the inquiry had been perfunctory and that the teachers doing the work were uninterested. Hall took pains to engage four experienced kindergarten teachers, to see to it that they were reasonably motivated, and to allow for cross-questioning—perhaps an early example of concern about the PCK of the interviewers and allowing for a degree of Socratic dialogue, or at least probing beyond the standard interview questions. According to Cairns (1983), they held small group interviews (*n* = 3) to make children feel at ease with the interview environment. The 134 items in Hall’s questionnaire were largely drawn from the same seven areas used in Germany; namely astronomy, mathematics, meteorology, animals, plants, local geography and miscellaneous (Glover & Ronning, 1987, p. 46). As in Lange’s studies, Hall found ‘a pronounced ignorance of the natural world’ particularly in city children and recommended that all children would benefit from time in the country (Hall, 1883, p. 255; in Young, 2016).

In pivotal research published in two books, Piaget (1929 and 1930) reported his investigations into European children’s views of the origin of the Sun and Moon, the movement of the clouds and heavenly bodies and the concept of Life, in children aged 2–13 years. He was against teachers presenting the Copernican (heliocentric) system to children as a hypothesis for the movement of the heavenly bodies because ‘the heliocentric view is so far removed from children’s conceptualisations of the earth-sun relation that it would be quite fruitless to teach young children this view’ (1930, p. 85). He criticised ‘tests’ in his 1930 paper because the method ‘does not allow a sufficient analysis of the results’ and it ‘falsifies the natural mental inclination of the subject or at least risks doing so’ (p. 3). He went on to say that: ‘The only way to avoid such difficulties is to vary the questions, to make counter-suggestions, in short, to give up all idea of a fixed questionnaire’ (p. 4). This one-to-one open-ended questioning technique came to be known as the ‘clinical method’. Piaget’s questions, according to his 1929 text, were determined in manner and in form, by the spontaneous questions actually asked by children of the same age or younger.

Claparède, in the introduction to Piaget (1926), summed up the ‘clinical method’ thus:

The clinical method is the art of questioning: it does not confine itself to superficial observation, but aims at capturing what is hidden behind the immediate appearance of things. It analyses down to its ultimate constituents the least little remark made by the child. It does not give up the struggle when the child gives incomprehensible answers but only follows closer in chase of the ever-receding thought, drives it from cover, pursues and tracks it down, till it can seize it and lay bare the secret of its composition. (Claparède, cited in Brearley & Hitchfield, 1966, p. xiv).

Bliss (1993) praised Piaget’s interview methodology, his rapport with children, and his attention to detail with the following words:

From a methodological point of view, Piaget established a tradition. He went into schools and talked informally to children; he usually devised some interesting activity or task for the child to do as the focus of the conversation; but above all he listened and valued what children said. This approach, known as the clinical method, has now been widely adopted, but unfortunately is sometimes not carried out with as much care as Piaget himself insisted on (p. 39).

As will become apparent in due course, most of the research conducted since Piaget’s early work, and reviewed here, has utilised Piagetian clinical interviews.

Shortly after World War II, in the USA, Oakes (1947) interviewed 153 children, aged 4–13 years, about their cosmologies and compared his results with all known work providing a valuable source. He found no evidence of gender mediation in his own studies of children’s cosmologies, nor in his extensive review of the literature extant at that time. More recent studies have continued to provide support for the view that boys and girls have similar, holistic-rather-than-fragmented, cosmologies which have features in common across cultures and ethnic groups (see Bryce & Blown, 2007 in due course).

Also in the USA, Haupt (1948, 1950) reported a discussion on the Moon between a teacher and her class of children, aged 6–7 years in 1948 (part 1). Interviews with the class individually in 1950 were reported in part 2. The first study involved free discussion prompted by ‘Let’s talk about the Moon’, analysis of the children’s responses yielding five categories. Each of these was subjected to an array of detailed questions which were used for the individual interviews in the second study. In his conclusion, Haupt stated that ‘It seems, sometimes, that imagination is *the* most important factor in children’s thinking’ and he judged that there was a need for further research into the roles of imagination ‘in various levels of children’s thinking’ (p. 233). This is something we have considered in detail from a quite different perspective incorporating neuro-scientific findings in Bryce and Blown (2021)—see later.

In the late 1970s, after a gap of over 40 years, Nussbaum and Novak (1976) used Piaget’s techniques to re-investigate children’s cosmological ideas, the individual interviews being supported by a structured questionnaire and various drawings and models as props. Working in the USA, they studied the Earth shape and gravity concepts of 26 American children, aged 8 years, using an Interview-Instruction-Interview research design; i.e. each child being given a structured interview patterned after Piaget’s clinical interview technique. They developed an *Earth Notions Classification Scheme* which has been of seminal importance. The interview procedure was developed with the aim of revealing the child’s version of the Earth concept, the final form of which was achieved through a developmental process consisting of several phases. In each phase, the form of the interview test was improved over the previous one in terms of number and variety of tasks, the quality of the drawings and the calibre of the questioning style. Each improvement revealed further differentiations in children’s notions. This led to further improvements, by suggesting better or completely new tasks, drawings and probing questions; and so on, iteratively. The basic tasks common to nearly all the assessment items involved *predicting* directions of imaginary free fall occurring at different points on a model of the Earth and *explaining* these predictions. Nussbaum and Novak observed that visual props were apt to provide the child with some cues that would interfere with the spontaneity and authenticity of his/her natural thinking (thereby risking the validity of the interview interpretation). They decided, therefore, to start the interview with a set of questions including ‘What is the shape of the Earth? How do you know that the Earth is round? …’ in the *absence* of any visual Earth model. Only after presenting these questions were a globe and pictures introduced into the interview (pp. 536–537). The presence or absence of such stimuli has proved controversial, as we shall see in due course.

In the same year, Za’rour (1976) interviewed 55 Christian and 55 Muslim children, aged 4–9 years in the Lebanon, about their concepts of phenomena such as rain, Sun, wind, Moon and falling bodies. [Za’rour’s text refers to *Moslems*, the normal term in use at that time.] He found little evidence of animism. Significantly more Christians perceived the Moon as having phases than Muslims (*c.f*. Bryce & Blown, 2013 for further considerations of how children perceive the shape and size of the Moon).

In a follow-up study, Nussbaum (1979) interviewed 240 children, aged 10–14 years, in Israel about their Earth shape and gravity concepts. His findings caused him to modify Nussbaum and Novak’s (1976) *Earth Notions Classification Scheme* merging two of the earlier notions and adding the notion of a two hemisphere Earth; the lower synonymous with ground; the upper with sky. The open-ended interview questions from the previous study were modified to a multiple-choice format with each of the four alternative answers presented by a drawing. The alternatives were designed so that each of them would represent at least one of the five notions described above and based on concrete answers offered by children in the previous study. The children were asked to explain their choices. At the start of each interview, the student was asked to draw a picture that would include the Earth, the sky, the Sun and the Moon, and some enlarged figures of people standing on the ground. This was done in order to obtain an evaluation of the child's conception of the Earth *before* they could be influenced by the multiple-choice format. The interviewer conversed with each child whilst he/she was constructing their own picture. Each half of the sample (cross-cut longitudinally) was questioned by a different trained interviewer and each individual interview lasted for 20–30 min. The child’s responses, choices and explanations were recorded immediately and were analyzed on the same day by his/her interviewer. In the conclusion to this well-planned and carefully executed study, Nussbaum stated that this Piagetian method ‘made possible a “penetration” into the children’s cognitive structure’ (p. 92).

Researchers Mali and Howe (1979, 1980) interviewed Nepalese children: 128, aged 8–12 years, from the Kathmandu valley, and 128, also aged 8–12 years, from the Pokhara valley, to ascertain their concepts of Earth. Using Nussbaum and Novak’s (1976) classification scheme, they found that Nepalese children had similar notions to American children but there was an age decrement of 4 years. Similar differences were found between the Nepalese groups, access to school being a critical factor in acquiring scientific knowledge. With respect to Piagetian tasks of conservation, seriation and classification, the researchers found that children from rural Pokhara equalled and at some points surpassed their urban counterparts from Kathmandu in conservation of area and weight at all ages. Mali and Howe argued that this was due in part to the rural children having experience in serving tea and other goods from their home stalls. Thus the comparative aspects of this study were unusually refined, the researchers focusing carefully on the important features of the cultural surroundings of the children being interviewed. The authors state in their 1980 paper that, contrary to their earlier findings on Nussbaum and Novak’s (1976) Earth concepts, the Nepalese children ‘were not retarded [*sic*]’ with respect to European children in conservation of area, weight and volume.

The 1980s saw several teams researching children’s cosmologies. In Israel, Nussbaum and Sharoni-Dagan (1981) used the Interview-Instruction-Interview design with 41 children, aged 7–8 years, to investigate methods of teaching scientific concepts of Earth. They advocated that teachers should actively encourage cognitive disequilibrium or dissonance as a strategy to enhance cognitive accommodation of more scientific concepts. The first ten questions of the interview plus the drawing task probed the child’s notion of the Earth as being a huge sphere ‘surrounded’ by cosmic space. Using three-dimensional props and drawn Earth models, the rest of the questions presented hypothetical situations on the Earth requiring the child to predict the direction of free fall of objects at different locations. The interview format and procedures were basically the same as those used previously (Nussbaum, 1979), with about half of the questions being open-ended and the other half being in a multiple-choice format. In the latter, each of the four alternatives was presented by a drawing. Children were asked to explain their choice and this added an open-ended component to each of the 14 multiple-choice items. Again, the question sequence was presented without any props at the beginning of the interview in order to obtain an evaluation of the child’s genuine conception of the Earth before he/she could become influenced by props and multiple-choice drawings. The strategy employed shows convincing links between sound research and ways in which teachers can act in accord with Ausubel’s (1968) dictum: ‘The most important single factor influencing learning is what the learner already knows. Ascertain this and teach him (*sic*) accordingly’ (p. vi).

From a comparative perspective, Klein (1982) interviewed 12 Mexican–American and 12 Anglo-American children, aged 7–8 years, in the USA, and discovered a poor understanding of Earth concepts in both groups. She emphasised the need to compare children of similar socio-economic background since cross-cultural studies often used dissimilar groups confounding linguistic, cultural and socio-economic factors, something better dealt with in some of the aforementioned pieces of comparative research.

Researchers Sneider and Pulos (1983) interviewed 159 US children, aged 9–14 years; classified their data using Nussbaum and Novak’s (1976) scheme; and compared the distribution of the notions held at each age level in their own and all previous studies. They developed *Earth Shape and Gravity Scales* which complemented the earlier scheme.

Treagust and Smith (1986) investigated Earth shape and motion, gravity and the Solar System with 24 children in Australia, aged 15 years, interviewed in small groups (*n* = 4). The purpose of the study was to examine secondary students’ understanding of the Solar System following a course of instruction and to identify misunderstandings and misconceptions. As part of their investigations into how students understood the motion of the planets, these authors use a series of interview cards representing fictional solar systems where the planets differed from our own, a strategy which has not found favour with others. Categories of misconceptions associated with gravity and the Sun’s source of energy were identified. Discussions were conducted with groups of 3 or 4 students, in three different schools, from classes who had completed the astronomy topic. Each interview lasted approximately 30 min, was tape recorded and later transcribed. Recommendations were made to improve the teaching of Solar System Astronomy in Australian schools.

Brewer et al. (1987) searched for evidence of cultural mediation of Earth concepts among 26 children, aged 6–12 years, in Samoa, whom they interviewed and conducted modelling sessions with clay. They found cases of children making ring-shaped models of the Earth which were believed to be influenced by the layout of houses in a Samoan village. Although valuable from a methodological and cross-cultural perspective, this study was overtaken by the substantial papers of Vosniadou and Brewer (1990, 1992, 1994).

Based on work with children in Greece and the USA, Sadler (1987) investigated concepts of day and night, the seasons and the phases of the Moon with 25 children, aged 15 years. His taped interviews were used in the award-winning documentary *A Private Universe* (Schneps & Sadler, 1989). The interviews partly informed Sadler’s study of 1414 US high school students’ astronomical misconceptions using multiple-choice tests (Sadler, 1992). Views that were incorrect but held by a large number of students were used as distractors.

Researchers Jones et al. (1987) interviewed 32 children, aged 9 and 12 years, in Australia, concerning their concepts of the shape, size and motion of the Earth, Sun and Moon (ESM). The children were questioned using a clinical interview technique and stimulus materials. A number of alternative views were shown to be held. The authors stated that: ‘use of a similar procedure with a group of children would seem to offer a powerful teaching methodology in which both teacher and students gain from the dialectic learning situation that is developed by this technique—apart from providing insights concerning children’s understanding’ (p. 43).

In England, Baxter (1989) carried out interviews with 20 children, aged 9–16 in an attempt to discern the notions used by them to account for easily observed astronomical events. Noting the development of these notions and historical parallels in astronomy, he found that early [learned] concepts ‘are not *exchanged* for the accepted theory’ (p. 506). The wider study of which the findings were part was intended to develop materials and approaches for teaching astronomy as part of the science curriculum of all pupils.

The 1990s saw an increasing focus on methodological improvements to the interviewing of children to ascertain their ideas in the field of astronomy. The concepts of 60 European-American children, aged 6–11 years in the USA and 90 children, aged 5–11 years in Greece were explored by Vosniadou and Brewer (1990) using a questionnaire in interviews that lasted from 30 to 45 min. These researchers used factual questions (e.g. ‘What is the shape of the earth?’, p. 610) and ‘generative’ questions (e.g. ‘If you were to walk for many days would you ever reach the edge of the earth?’, p. 610), arguing that the latter would better reflect children’s conceptual knowledge. They observed that children in both cultures had difficulty in understanding that the shape of the Earth was spherical but that they consistently used a limited number of alternative *mental models* to explain their cosmologies (each with elements of intuitive and scientific concepts).

The same researchers interviewed 60 children in the USA, aged 6–11 years, and developed a classification taxonomy of concepts of Earth shape and habitation (Vosniadou & Brewer, 1992). Five mental models of the Earth were identified: rectangular, disc, dual earth, hollow sphere and flattened sphere. The methodology followed that described in the 1990 paper. Children were asked 15 questions about the shape of the Earth from a 48-item questionnaire. Follow-up questions were used to clarify those responses which the researchers could not understand. Children were asked: ‘tell us more about it’ or the last part of a child’s response was repeated as a question. In a few cases, when the researchers could not understand what the children were telling them, they were forced to engage in more extensive questioning (pp. 543–545).

For their following study, Vosniadou and Brewer (1994) interviewed 60 children aged 6–11 years; also in the USA, the researchers developed a classification taxonomy of concepts of the causes of the day/night cycle. The methodology followed that in the1990 paper described above. Thirteen questions about children’s ideas about the disappearance of the Sun at night, the movement of the Moon, explanations of the day/night cycle and the disappearance of the stars during the day were selected from their questionnaire on astronomy concepts. Detailed notes were made of children’s responses and the interview recorded using a tape-recorder. The scoring was done later on the basis of both the transcribed data and the experimenter’s notes. The results showed that the majority of children used a small number of relatively well-defined mental models of the ESM consistently to explain the day-night cycle.

Comparative investigations continued to figure in the 1990s. Nakashima (1993) interviewed 80 Japanese children, aged 6–9 years, investigating their concepts of Earth shape; and 128 children, aged 6–9 years, investigating Earth shape and gravity. The study focused on how the students tried to inter-relate their own, informal observational knowledge with conflicting ‘knowledge from scientific information’, in most cases with little success. According to the author, achievement required explicit instruction.

Samarapungavan et al. (1996) questioned 38 Indian children, aged 5–8 years, individually in interviews lasting approximately 45 min and compared their results with those of their earlier American studies (Vosniadou & Brewer, 1990, 1992, 1994). They found more disc-shaped-Earth cosmologies in Indian children vs. American children, which they took as evidence in support of their hypothesis that children’s cosmologies are constrained universally and culturally. Firstly, by universal first-order constraints such as that the Earth is flat and supported; and secondly by cultural constraints such as notions of the Earth’s shape and location relative to the Sun and Moon limiting explanations of the day-night cycle. Many Indian children borrowed the idea that the Earth is supported by an ocean or a body of water from folk cosmology. The questionnaire used was a modified version of that developed by Vosniadou and Brewer (1990, 1992, 1994). Some questions required only verbal responses; others required the children to explain their responses with clay models that they made or with pre-made Styrofoam models that they selected.

Diakidoy et al. (1997) interviewed 26 US American-Indian children, of Lakota/Dakota ethnicity, aged 6–11 years, to ascertain their models of the shape of the Earth and the causes of the day/night cycle. The methodology followed that described in Vosniadou and Brewer (1990). A 45-item questionnaire was used with 18 questions on Earth shape, 10 questions on the day/night cycle and 17 questions on Earth's motion and the stars. The results indicated that the children used a small range of relatively well-defined models of the Earth and the day/night cycle similar to those found in previous studies. However, the children preferred a hollow sphere model, in keeping with Lakota/Dakota mythology. Younger children also used some novel animistic explanations of the day/night cycle. They concluded ‘that while the process of knowledge acquisition in astronomy follows a similar path in all children regardless of cultural variables, cultural cosmology influences both the specific models constructed as well as the modes of explanation provided for astronomical phenomena’ (p. 159).

In Sharp (1999), the author interviewed twenty-five 7-year-olds, one-to-one, in England about the Earth in space and other areas of astronomy. Probes containing both verbal and non-verbal techniques were incorporated. These included open conversations, discussions about specific instances and events, the manipulation of physical props, drawings, word association and picture recognition (Osborne et al., 1985; White & Gunstone, 1992). Responses were collected in writing, recorded on tape and interpreted in terms of their content, accuracy, language, logic and reasoning, sources of information and consistency. Responses were also compared alongside those from previous studies (Vosniadou & Brewer, 1992). The researcher concluded that focusing on Earth shape and gravity alone, however, may have resulted in an underestimation of children's other abilities and learning potential in this field.

In the early 2000s, children’s cosmologies research, internationally, continued to see efforts to advance interview methodology. Researchers Vosniadou et al. (2001) interviewed a class of 5^th^ and 6^th^ grade students in Greece to ascertain children’s concepts of mechanics (force and energy) as part of a broader study of astronomy. In addition to pre- and post-tests, interviews were used to clarify some of the questions regarding children’s understanding that could not be answered by the analysis of their responses in the written tests. The interview, which was really a discussion with the interviewer who was also the teacher, was a situation where the students were prompted to express their opinions, and were helped by the teacher through hints to re-evaluate their answers, and to provide more information. Thus, the interviews tested the students more at the *Zone of Proximal Development* (ZPD^3^) (Vygotsky, 1978) than the post-tests did.

In the same year, researchers Schoultz et al. (2001) published their findings from interviews with 25 children, aged 6–11 years in Sweden, having set out to ascertain the effectiveness of lessons based on socio-cultural theory. In contrast to Vosniadou et al., they used cultural artefacts such as globes and the concept of countries as a focus, to investigate children’s concepts of the shape of the Earth and gravity from a ‘situated and discursive perspective’. That is, Schoultz et al. considered it proper to ‘tune’ the subjects in to what the interviewers were interested in asking about; focusing their minds on what sorts of things were to be thought about in front of them. They concluded that the globe served as ‘a discursive structure with clear boundaries’ which enabled all of the participants to express scientific concepts rather than intuitive ones. As raised in point (d) of our introduction, their view of the value of verbal responses in interviews reflects their socio-cultural perspective encapsulated in the statement: ‘We shall not favor the assumption that what is said in an interview situation is a reflection of conceptual content in the mind of the individual’ (p. 109).

Following this, and again in Sweden, Ivarsson et al. (2002) investigated conceptions of the shape of the Earth and gravity through a study of maps and countries with 18 children, aged 7–9 years. They found no evidence of children having mental models (see Vosniadou & Brewer, 1992, 1994). Such ‘constructs’ were regarded as a product of the interview methodology, so that when cultural artefacts such as maps are introduced these intuitive notions effectively ‘disappear’. This argument is contested, not least by Nussbaum and Novak (1976); Vosniadou et al., (2004 and 2005); Blown & Bryce (2006); and others, who consider the early presentation of artefacts like globes leads to conflicts with incompatible prior/intuitive knowledge in many children; the resulting dialogue masks what that understanding actually is.

Nobes et al. (2003) interviewed 167 children (82 Asian Gujarati and 85 Caucasian), aged 4–8 years, in East London to ascertain their concepts of the shape of the Earth. Children had to select from a set of plastic models and answer forced-choice questions without having to explain or justify their responses, and the three fieldworkers involved in the research ‘had some knowledge of previous work in this area …’. The authors found no significant differences between cultures once allowance was made for variations in language skills. Their results indicated that children's knowledge was fragmented rather than coherent [aligning themselves with diSessa (1988) rather than Vosniadou and Brewer (1992) and Blown & Bryce (2010)].

Siegal et al. (2004) interviewed 59 children, aged 4–9 years, in Australia, and 71 children, age 4–9 years, in the UK, and found the Earth concepts of Australian children more advanced than those of English children. Due to early instruction in this domain in Australian schools, this did not surprise Siegal et al. and they concluded that coherence vs*.* fragmentation in children’s concepts is a reflection of the timing of culturally transmitted information as well as the questioning methods used in research.

Working in Athens, Vosniadou et al. (2004) investigated how methods of questioning affected children’s responses regarding the shape of the Earth and the day/night cycle. Seventy-two children from Grade 1 and Grade 3 in a middle-class elementary school were tested individually at school by two experimenters, either by an open method or by a forced-choice method of questioning. The interviews lasted approximately 15–20 min for each child. Different results were obtained from the two methods of testing, suggesting that they tapped different forms of knowing and different ways of reasoning. The open questioning replicated Vosniadou and Brewer’s (1992) findings, i.e. that the majority of the responses were consistent with a small number of internally consistent mental models. The forced-choice method of questioning resulted in more scientifically correct responses, but also fewer internally consistent ones. The authors concluded that the forced-choice method of questioning, together with the presentation of the spherical model of the Earth, could inhibit the generation of internal (mental) models.

In a follow-up study, Vosniadou et al. (2005) interviewed 44 children, aged 6–7 and 8–9 years, again in Greece, to determine their concepts of Earth shape. They found that the use of globes as interview props inhibited the generation of mental models. ‘It appears that in the absence of an external, cultural model, children can form internal representations which they can distort in ways that make them consistent with their prior knowledge. But, when the cultural artifact is present, such distortions are not possible with the result that children end up with internally inconsistent patterns of responses’ (pp. 333–334).

In longitudinal studies, spanning several years between 1987 and 2000, Bryce & Blown (2006) investigated the cultural mediation of children’s thinking about the Earth using a Piagetian interview technique designed to elicit responses from children from all ‘levels’ of their conceptual organisation (intuitive, cultural and scientific). An interview guide designed to cover the syllabus of astronomy and Earth science topics common to children in New Zealand (abbreviated NZ) and China at each level was used. The instrument was originally written in English which was translated into *Hanyu* (Mandarin) to assist trained interpreters in China. This was complemented by Socratic dialogue to clarify responses when appropriate. Close scrutiny of the research literature had revealed that some strategies used in the past to probe children’s ideas have been influenced by the background of the interviewer, either in the design of their questions or in the use made of concrete props (e.g. of the Earth’s shape). This tended to obscure the degree of cultural influence of those interviewed. Central to this research therefore was the development of an interview method (‘instrument attunement’) which was flexible, culturally adaptable and could be tuned to the response level of the child. The participants included 129 boys, 113 girls from China and 213 boys, 227 girls from NZ. The methodology utilising the children’s own observations of the Sun and the Moon led into discussion of the motion and shape of the ESM. The 2^nd^ author (from NZ himself) spent considerable time in both the NZ and the Chinese school communities in order to become familiar to the subjects in their schools. Surprisingly, the development of children’s concepts was found to be remarkably similar within the three main ethnic groups (China Han, NZ European and NZ Māori) in the two cultures (China and NZ). Cases of cultural mediation were detected but these could be assimilated into a common taxonomy of cosmological concepts for all participants.

Further to the above (and published in Blown & Bryce, 2006), children’s cosmologies were investigated over a 13-year period, using multi-modal, in-depth interviews with 686 children (217 boys, 227 girls from NZ and 129 boys, 113 girls from China), aged 2–18. The procedure followed that of Bryce and Blown (2006) using questions from a comprehensive interview guide supported by Socratic dialogue. Children were interviewed whilst they *observed* the apparent motion of the Sun, the motion and phases of the Moon and features of the Earth; *drew* their ideas of the shape and motion of the ESM, and the causes of daytime and night-time; then *modelled* them using play-dough, which led into *discussion* of related ideas. Models of Earth shape were not introduced to clarify children’s responses—in contrast to Vosniadou and Brewer’s (1990) approach where children were asked to select an Earth shape from a range of models, the choice of which compared favourably with what they had said in generative dialogue. Although this paper supports Vosniadou and Brewer’s claim that children have coherent notions about the shape of the Earth, the introduction of models was considered to inhibit rather than assist the investigative process. The interviews revealed that children’s cosmologies were far richer than previously thought and surprisingly similar in developmental trends across the two cultures. There was persuasive evidence of three types of conceptual change: a long-term process (over years) similar to weak restructuring; medium-term processes (over months) akin to radical restructuring; and a dynamic form of conceptual crystallisation (often in seconds) whereby previously unconnected/conflicting concepts gel to bring new meaning to previously isolated ideas. The interview technique enabled the researchers to ascertain children’s concepts from intuitive, cultural and scientific levels, and supported the argument that children have coherent cosmologies which they actively create to make sense of the world rather than fragmented, incoherent ‘knowledge-in-pieces’.

Although the current paper has focused on interviews and verbal language as a primary source of knowledge, our own technique has been essentially multi-modal, including children’s drawings and play-dough modelling. We have also reported the multiple sources of astronomy knowledge utilised by children (including peers), knowledge of which informed interview design and technique (see Blown & Bryce, 2020). As Espinoza (2005) suggests, referring to US students in an experimental study, teachers should make greater use of thought-experiments mediated by student peers to overcome resistance to accepting scientific interpretation of Newtonian concepts of force and gravity (see Blown & Bryce, 2013; Bryce & Blown, 2013). Espinoza’s design included opportunities for students to respond to questions about gravity and pendulum motion by drawing and by video. The latter sessions were conducted by a fellow student who put the questions: ‘In the laboratory activity, a student volunteer described the situation, demonstrated the motion, and then asked the same questions as those in the pencil-and-paper task.’ (pp. 276–277), thus offering a degree of collaborative learning (see Bryce & Blown, 2012). See reference to the use of pendulums in our discussion of observational astronomy below.

As reported in the first of two papers, Panagiotaki et al. (2006a) tested the influence of question type (open vs. forced-choice questions) and medium (drawings vs. 3-D models) on the scores of a sample of 59 6-year-olds in England. They found that the use of drawings and open questions increased the apparent incidence of naïve mental models, and the combination of physical 3-D models plus forced-choice questions elicited more scientifically correct responses (as well as higher proportions of scientific and inconsistent mental models than the combination of drawings and open questions). The researchers argued that ‘…. children know more about the earth than the mental model theorists claim, and that naïve mental models of the earth are largely artifactual’ (p. 353). In their second paper, Panagiotaki et al. (2006b) describe the use of one open and eight multiple-choice questions (with varied numbers of response alternatives ranging from 2 to 4 for written items and 7 for the items involving 3-D models). They concluded that only 10% of the children showed any evidence of naïve mental models. However, as the writers acknowledge: drawing, model-making or answering open questions require free-recall and imagination, as against the easier, recognition demands of forced-choice tasks.

In the UK, Sharp and Sharp (2007) conducted a ‘quasi-experiment’ with 31 children, aged 9–11, learning about astronomical ideas in two vertically grouped classes in an English primary school located in a mixed socio-economic catchment area. (Children in the control group were taught the ideas later.) The class teacher of the experimental group was described as ‘an enthusiastic, 36-year-old male practitioner with 15 years teaching experience and a positive attitude and constructivist orientation towards primary science’ (p. 376), i.e. with good subject and pedagogic content knowledge. The children in the experimental group engaged in a wide variety of illustrative, investigative and problem-solving activities: reading about astronomy and conducting their own research; preparing their own encyclopaedia of space using a multi-media authoring package; working with concrete scientific models; observing the sky at night as parentally supervised homework; and with parents and children participating in an evening trip to a local observatory. Interviews pre- and post-intervention of an hour’s duration were recorded.

Continuing their work in Greece, Skopeliti and Vosniadou (2007) interviewed 84 children aged 6 and 8 years individually, using a questionnaire similar to that used by Vosniadou and Brewer (1992) supported by a map and globe. In a pre-test, children were asked to make drawings and play-dough models of the Earth and to indicate where people live. The sample was split, one half given a globe, the other a map, and both asked further questions. The researchers found that the post-test influenced what children then said, relying on their incompatible prior knowledge. (‘… presentation of the globe caused a dramatic change in children’s responses regarding the shape of the Earth with most children abandoning their previous representation of the earth and adopting the culturally accepted representation’.) The researchers concluded that ‘the use of an external representation is not an act of “direct cultural transmission”, but a constructive process during which the information that comes from the culture is interpreted and influenced by what is already known’ (p. 244).

As part of the comparisons between children’s intuitive/informal scientific knowledge and their later achievements and attitudes in school science, Bryce & Blown (2007) examined the gender-related findings from in-depth interviews with 119 boys and 121 girls, ranging from 2 to 12 years, in China and NZ. The interview used questions from an extensive interview guide complemented by Socratic dialogue as detailed in Bryce and Blown (2006). The questions and the interview framework were designed to maximise flexibility to permit children to share their cosmological concepts over a wide age range. By comparing boy/girl cosmological concept categories and by tracking their developmental trends by age, statistical evidence revealed the extent of the similarities within and across these diverse cultures. The findings reinforceed those from the authors’ previous studies (as above) and provide support for the view that boys and girls have similar, holistic-rather-than-fragmented, cosmologies which have features in common across cultures and ethnic groups.

In a series of three studies, Wilhelm (2009a, b; 2014) investigated US children’s concepts of the Moon and shadows. Topics included the Moon’s changes in appearance; the Moon’s size; the Moon’s distance from Earth; and the source of illumination of the Moon. Through Piagetian interviews, children’s ideas about the cause of lunar phases and the nature of shadows were ascertained and clarified. It was found that children gained astronomical knowledge from a variety of sources including family, their own observations and experience. In her later study, Wilhelm investigated gender differences in astronomical learning particularly lunar phases. With a pre-test/post-test design utilising a Lunar Phases Concept Inventory and a Geometric Spatial Assessment, she found that males scored significantly higher than females on the science domain of assessment and females made significant gains in the mathematics domain. (In Bryce & Blown, 2007 studying New Zealand and Chinese children, we reported girls’ superior ability to visually represent their cosmologies and boys’ greater awareness of gravity; and in Blown & Bryce 2020 we identified teachers, parents and librarians as major sources of astronomical knowledge, as well as children’s “own observations” as important sources.)

In Blown and Bryce (2010), we describe the study of 345 young people over a 10-year period using a multi-media, multi-modal methodology in a research design where survey participants were interviewed three times and control subjects were interviewed twice. The interviews used standard questions supported by Socratic dialogue when opportune as detailed in (Bryce & Blown, 2006). Each interview session took between 40 and 120 min overall (with appropriate breaks between studies) depending on the age of the child. An interview guide designed to cover the syllabus of astronomy and Earth science topics common to children in NZ and China at each level was used. Five hypotheses were confirmed rejecting the *knowledge*-*in*-*pieces* argument in favour of *conceptual coherence*: (a) conceptual coherence shown as patterns of high *correlation* of concept representations between the media used to assess subjects’ understanding *within* a survey, as well as (b) coherence revealed as *consistency* of those concepts across modalities; (c) enhanced conceptual understanding and skill through repeated interviews *across* (longitudinal) surveys, as young people develop their knowledge; (d) cultural similarity in subjects’ representations of *basic static concepts* (e.g. the shape of the Earth); and (e) improved understanding of *basic dynamic concepts* (e.g. the motion of the Earth) and *complex dynamic concepts* (e.g. seasons and eclipses), interpreting a concept as a skill (*c.f.* Barsalou, 2003).

In the same year, Hannust and Kikas (2010) describe a longitudinal study carried out in Estonia, where the investigators followed the development of Earth concepts of 143 children, aged 2–3 at the start, for 3 years. The children were interviewed at annual intervals utilising the same questions (based on Vosniadou & Brewer, 1992) on each occasion. Hannust and Kikas reported that ‘in most cases young children’s knowledge was fragmented and accurate knowledge was often expressed alongside inaccurate/ synthetic ideas’ (p. 164). They argued that children needed to know scientific facts before they start taking the global perspective when describing the world. ‘When faced with ambiguous open questions, children often experience difficulties that can induce them to change the types of answers they provide’ (p. 164). Most of the ‘synthetic answers’ found in the study were attributed to the nature of the tasks used in the study.

Also in the same year, Plummer, Wasko and Slagle (2011) investigated elementary pupils’ explanations for the daily patterns of the apparent motion of the Sun, Moon and stars, interviewing 24 US children, aged 8–9 years. At Grade level 3 in the USA, national standards indicate that such children ‘should learn to use the Earth’s rotation to explain daily celestial motion’ (Abstract, lines 4/5). The research indicated that about half of the sample were working from naïve mental models, the other half were using more scientific explanations but far less frequently. An instructional program using computer simulations and models suggested that pupils of this age could be helped to move between Earth-based and heliocentric frames of reference in their thinking.

Fre’de, Nobes, Frappart, Panagiotaki, Troadec and Martin (2011) studied the influence of methods of questioning and analysis on the interpretation of children’s conceptions of the Earth. They interviewed 178 French children, aged 5–11 years, comparing forced-choice questions with open-ended questioning to ascertain whether their knowledge was coherent or fragmented. Children were interviewed individually for about 30 min in a quiet area of their school with rapport established and explanations given. All protocols were scored twice by two independent judges. Agreement reached 100% for the forced-choice conditions and 87% for the open conditions. All disagreements were resolved through discussion (p. 437). The study found that forced‐choice questions resulted in higher proportions of scientific answers than open questions, and children appeared to have naïve mental models of the Earth only when the mental model coding scheme was used (thus supporting the fragments of knowledge argument and that naïve mental models of the Earth are methodological artefacts).

Knowledge in astronomy across a broad spectrum of ages and experience in China and NZ was investigated in Bryce and Blown (2012) article on ‘novices’ vs. ‘experts’. There were 960 participants in all, aged 3–80 years, including 68 junior school pupils; 68 primary school pupils; 111 middle school students; 109 high school students; 79 physics undergraduates; 60 parents; 103 pre-service primary teachers; 131 pre-service secondary teachers; 72 primary teachers; 78 secondary teachers; 50 amateur astronomers and astronomy educators; and 30 astronomers and physicists; with approximately equal numbers of each group in both cultures; and of boys and girls in the case of children. The methodology utilised Piagetian interviews with three media (verbal language, drawing, and play-dough modelling), as described in Bryce and Blown (2006) in the case of children; and a written questionnaire for adults. A combination of closed and open questions to afford different forms of reasoning at all levels of experience was used. Closed *How*? questions investigated scientific knowledge anticipating simple statements about phenomena (e.g. How the Earth moves). Whereas open-ended *Why*? questions invited more complex explanations of the cause of phenomena (e.g. Why the Earth moves). The results showed that expertise (as scientific knowledge and conceptual skill) is a process of gradual acquisition from childhood to adulthood and from novice to expert.

Venville et al. (2012) carried out a detailed interview study with a small group of children, aged 3–8 years, eight in the USA, two in Australia. To seek information about possible social and cultural influences on their knowledge, parents were also interviewed. The authors detected evidence of both the *framework theory* perspective and the *knowledge in pieces* perspective in student knowledge: The children ‘… provided [us] with a kaleidoscope of ideas about the Moon and the social and cultural experiences that influenced their ideas…’ (p. 745). The writers stated their wish for further similar case studies with children of different ages and cultures.

Two papers by Tao et al., (2012, 2013) describe studies of 54 children, aged 8 years, in China, and 54 children, aged 8 years, in Australia, who were interviewed about their concepts of Earth shape, gravity, day/night and the seasons following the questions used by Vosniadou and Brewer (1992, 1994). The children were drawn from schools in districts of high, medium and low socio-economic status in both countries. A science quiz was used to assess scientific understanding and in-depth interviews used to further explore conceptual understanding. The researchers reported that: ‘Most children were not sure about the rotation of the Earth, the Sun and the Moon. Some thought the Sun and Moon stayed in the sky, and the Earth stayed in the middle and rotated; others explained that the Earth rotated around the Moon, and the Moon rotated around the Sun’ (2012, p. 892). Note. The use of ‘rotated’ as synonymous to ‘revolved’ is not uncommon in the literature (see Blown & Bryce, 2017, discussion on p. 651). In a further study reported in the 2013 paper, the same authors interviewed 18 children, age 8 years, and 20 children, aged 12 years, in China; and 18 children, aged 8 years, and 18 children, aged 11 years 6 months, in Australia about their concepts of Earth shape, gravity, day/night and the seasons. They found that regardless of culture, children of similar age held similar concepts about the Earth, with Year 3 pupils more likely than Year 6 pupils to demonstrate intuitive concepts of a round and flat Earth, whilst Year 6 pupils were more likely to demonstrate consistent understandings of a spherical Earth. The authors state that ‘The findings supported the universality of entrenched presuppositions hypothesis. Cultural mediation was found to have a subtle impact on children’s understanding of the Earth’ (2013, p. 253).

An article by Blown and Bryce (2013) examined the continuity of thought-experiments about gravity throughout the ages and was used to contextualise a set of interviews with 247 children in NZ and China designed to ascertain their ideas about falling objects. The sample included 68 pre-school pupils, 68 primary school pupils, 56 middle school students and 55 high school students, with approximately equal numbers in each group and of boys and girls in each group in each culture. The methodology was as described in Bryce and Blown (2006). This included a series of three multimodal thought-experiments to probe concepts of gravity. The first involved drawing and modelling ‘Self’ and ‘a Friend on the other side of the world’ throwing and dropping balls, describing the path of the balls, and explaining why the balls moved as they did. The second scenario had ‘Self’ and ‘Friend’ placing drink bottles (part full, with the tops off) on the surface of the Earth, explaining in each case what would happen to the water and why it would happen. And the third thought-experiment entailed ‘Self’ dropping a ball into a deep hole through the Earth, describing what would happen to the ball, and why it would happen. From a cross-age perspective, it was found that young children displayed an intuitive sense of gravity which developed with age as a result of learning and experience in close association with concepts of Earth Shape and Earth Motion. From a cross-cultural viewpoint, the development of these concepts was found to be similar in China and NZ (cultures where teachers generally hold a scientific world view). Overall, taking into account both developmental and cultural strands, the results supported the argument that children’s concepts of the Earth and gravity are coherent, not fragmented ‘knowledge-in-pieces’.

In Bryce and Blown (2013), 248 children aged 3–18 years (119 from China and 129 from NZ) were interviewed using elements of both constructivist and socio-cultural methodologies to research children’s concepts of the shape and size of the ESM. The study was based on an ethnological, cross-cultural, longitudinal design utilising one-to-one Piagetian clinical interviews incorporating Socratic dialogue (as described in Bryce & Blown, 2006). The interviews were held in a setting with the interviewer as an accepted member of the culture and usually in a social setting in that other children and adults were not excluded from visiting and observing experiments. The interviews investigated children’s concepts of the Motion of the Earth through observation of changes in the shadow of a vertical shadow stick, followed by them being asked to draw the motion of the Earth. The children then drew and modelled the shape of the ESM and compared their sizes. The understanding which young people display during interactions intended to solicit their knowledge are very much a reflection of the sensitivity of the questioning they encounter, the ways in which they are allowed to show their understanding and of course the nature and extent of the relevant experiences they have had in their education to that point. ‘Testing’ is considered to be very much second best to ‘interviewing’ but the latter requires time, familiarization and acceptance of the researcher by the person whose ideas we seek to understand (particularly relevant in cross-cultural research).

Interest in children’s cosmologies continued internationally as shown by Saçkes, Smith and Trundle’s (2016) case study involving 56 children aged 4–5 years (27 in the USA, 29 in Turkey) using semi-structured, individual interviews. The authors concluded that the preschoolers were able to make comparable observations of the sky, consistent with the framework theory of developing knowledge. The better representation of science concepts and skills in US early education programs did not confer advantage to the performance of US over Turkish children.

The article by Bryce and Blown (2016) notes the convergence of recent thinking in neuroscience and grounded cognition regarding the way we understand mental representation and recollection: ideas are dynamic and multi-modal, actively created at the point of recall. Also, neurophysiologically, re-entrant signalling among cortical circuits (Dresp-Langley, 2012; Edelman, 2005) allows non-conscious processing to support our deliberative thoughts and actions. The qualitative research described in this paper examined the exchanges occurring during semi-structured interviews with 360 children aged 3–13, including 294 from NZ (158 boys, 136 girls); and 66 from China (34 boys, 32 girls) concerning their understanding of the shape and motion of the ESM. The standard questions from the interview guide being supported by questions seeking clarification of ideas through Socratic dialogue as in Bryce and Blown (2006). In particular, the research focus was on the switching taking place between what is said, what is drawn and what is modelled. The evidence was supportive of Edelman’s view that memory is *non*-*representational* and that concepts are the outcome of perceptual mappings, a view which is also in accord with Barsalou’s (2003, 2008) notion that concepts are simulators or skills which operate consistently across several modalities. Quantitative data indicated that the dynamic structure of memory/concept creation was similar in both genders and common to the cultures/ethnicities compared (NZ European and NZ Māori; China Han). Also, it was evident that repeated interviews in this longitudinal research led to more advanced modelling skills and/or more advanced shape and motion concepts.

The research reported in Bryce and Blown (2017) investigated the everyday and scientific repertoires of children involved in semi-structured, Piagetian interviews carried out to check their understanding of dynamic astronomical concepts like daytime and night-time. The methodology followed that of (Bryce & Blown, 2006) utilising a comprehensive interview guide of standard questions on basic astronomy complemented by Socratic dialogue. The research focused on the switching taking place between embedded and disembedded thinking (see Donaldson, 1978); on the imagery which subjects referred to in their verbal dialogue and their descriptions of drawings and play-dough models of ESM; and it examined the prevalence and character of animism and figurative speech in children’s thinking. Modified ordinal scales for the relevant concept categories were used to classify children’s responses and data from each age group (with numbers balanced as closely as practicable by culture and gender). Although in general there was consistency of dynamic concepts within and across media and their associated modalities in keeping with the theory of conceptual coherence (see Blown & Bryce, 2010; Bryce & Blown, 2021), there were several cases of inter-modal and intra-modal switching in both cultures. Qualitative data from the interview protocols revealed how children switch between everyday and scientific language (in both directions) and use imagery in response to questioning. The research indicated that children’s grasp of scientific ideas in this field may ordinarily be under-estimated if one only goes by formal scientific expression and vocabulary.

Greek students’ misconceptions about the day/night cycle after reading a science text were investigated in the study by Vosniadou and Skopeliti (2017). Further, 99 children; 50 aged 8; 49 aged 10 attending the same school in a middle-class Athens suburb sat a written pre-test where they had to (a) draw a picture of a person living on the Earth when it is daytime and when it is night-time and (b) write an explanation of day and night. The researchers then interviewed ten pupils aged 8, and ten aged 10 (testing and interviews took place in a small interview room in the children’s school). The results convinced the authors that initial explanations ‘continued to exist in the conceptual repertoire of the reader, and it is not difficult even for young children to retrieve it from memory and use it to create a situation model of the initial text’ (pp. 20–21). They also found that children had cultural explanations for the day/night cycle long before learning the scientific view, and that these explanations appeared to ‘co-exist with scientific ones even after conceptual change had been achieved’ (p. 21)—bringing into question what is meant by ‘conceptual change’.

In the study by Blown and Bryce (2020), the semi-structured, multimedia interviews described previously Bryce and Blown (2006) were used with 538 children (125 boys and 145 girls in NZ, 144 boys and 124 girls in China). These were augmented using questionnaires with 80 parents, 65 teachers and 5 local librarians. Together, these were used to investigate the sources of children’s astronomy knowledge, focused on their understanding of daytime and night-time and the roles played by the Sun and Moon in creating familiar events. The analysis (a) considered how teachers, parents and librarians (libraries, books) continue to be major sources of scientific knowledge despite the rise of electronic media (the Internet); (b) identified the extent to which folklore—both local and imported by migration—was an important source of that knowledge; and (c) showed how metacognitive bootstrapping and a growing awareness of co-existing everyday and scientific repertoires of knowledge resulted from divergent sources. Children frequently revealed their sources of information during the interview without prompting. In other cases, they were asked where they learned about the ESM.

Finally, Bryce and Blown (2021) followed the same interview methodology as in the 2018 paper described above, allowing about 1 hour per interview, 141 children aged 3–12 made up of 73 from NZ (36 boys and 37 girls) and 68 from China (34 boys and 34 girls). At the end of their interview, children were asked the following questions about imagery: (1) *When I asked you about the Earth*, *Sun and Moon did you think of any stories you have been told about them*? (2) *Did you see any words* (*in your imagination*)? (3) *Did you see any pictures* (*in your imagination*)? (4) *Did you see anything moving* (*in your imagination*)? (5) *Where did your ideas come from*? (6) *What were you thinking of*? The paper explored the dynamic nature of memory now accepted by neuroscientists who emphasise:

- Its creative (in contrast to its reproductive) character; and therefore
- Challenge the representational connotation often implicit in cognitive analyses of what children say when remembering; and
- Cast serious doubt on the common-place presumption that recall is akin to the extraction of ideas from a mental data-base.

The study re-affirms the merits of sensitive clinical interviewing. When used by an experienced researcher in conjunction with Socratic dialogue and triangulated with children’s drawings and models, it can yield valid data about children’s knowledge (as researchers like Vosniadou and her colleagues have productively demonstrated).

The research described in the preceding historical review of investigations into Children’s Cosmologies are listed by Researcher, Year of Publication and Topics in Table 1.

**Table 1:** Studies of children’s cosmologies showing researchers, year of publication of research and topics

Topics							
		Shape of Earth and/or Sun and/or Moon/phases of Moon	Gravity of Earth and/or Sun and/or Moon or falling objects	Habitation of Earth and Identity with Earth	Motion of Earth, Sun and Moon Seasons/ Eclipses	Day and Night	Location of Earth, Sun and Moon in Space/ Solar System
Researchers	Year						
Lange	1879	Yes	Yes	Yes	Yes	Yes	Yes
Hall	1880–83	Yes	Yes	Yes	Yes	Yes	Yes
Piaget	1929–30	Yes	Yes	Yes	Yes	Yes	Yes
Oakes	1933–35	Yes	Yes	Yes	Yes	Yes	Yes
Haupt	1948–50	Yes	Yes	Yes	Yes	Yes	Yes
Nussbaum & Novak	1976	Yes	Yes	No	No	No	No
Za’rour	1976	Yes	Yes	No	Yes	No	No
Nussbaum	1979	Yes	Yes	Yes	No	No	No
Mali & Howe	1979–80	Yes	Yes	Yes	No	No	No
Nussbaum & Sharoni-Dagan	1981	Yes	Yes	Yes	No	No	No
Klein	1982	Yes	No	Yes	Yes	Yes	Yes
Sneider & Pulos	1983	Yes	Yes	Yes	No	No	No
Treagust & Smith	1986	Yes	Yes	No	Yes	No	Yes
Brewer et al	1987	Yes	Yes	Yes	Yes	Yes	Yes
Sadler	1987–1992	Yes	Yes	Yes	Yes	Yes	Yes
Jones, Lynch & Reesink	1987	Yes	Yes	Yes	Yes	Yes	Yes
Baxter	1989	Yes	Yes	Yes	Yes	Yes	Yes
Nakashima	1993	Yes	Yes	No	No	No	No
Vosniadou & Brewer	1989–94	Yes	Yes	Yes	Yes	Yes	Yes
Samarapungavan et al	1996	Yes	No	No	No	Yes	No
Diakidoy et al	1997	Yes	No	No	No	Yes	No
Sharp	1999	Yes	Yes	Yes	Yes	Yes	Yes
Vosniadou et al	2001	Yes	Yes	Yes	No	No	No
Schoultz et al	2001	Yes	Yes	Yes	No	No	Yes
Ivarsson et al	2001	Yes	Yes	Yes	No	No	Yes
Vosniadou et al	2004, 2005	Yes	Yes	Yes	Yes	Yes	Yes
Nobes et al	2003	Yes	Yes	Yes	Yes	Yes	Yes
Siegal et al	2004	Yes	Yes	Yes	Yes	Yes	Yes
Panagiotaki et al	2006a,b	Yes	Yes	Yes	No	No	No
Sharp & Sharp	2007	Yes	Yes	Yes	Yes	Yes	Yes
Skopeliti & Vosniadou	2007	Yes	Yes	Yes	No	No	No
Wilhelm	2009a,b, 2014	Yes	No	No	Yes	No	Yes
Hannust &Kikas	2010	Yes	Yes	Yes	No	No	No
Plummer et al	2011	Yes	No	No	Yes	Yes	No
Fréde et al	2011	Yes	No	Yes	No	No	No
Venville et al	2012	Yes	No	No	Yes	Yes	No
Tao et al	2012, 2013	Yes	Yes	Yes	Yes	Yes	No
Saçkes et al	2016	Yes	No	No	Yes	Yes	No
Vosniadou & Skopeliti	2017	Yes	No	Yes	Yes	Yes	No
Blown & Bryce	2006–2020	Yes	Yes	Yes	Yes	Yes	Yes

## Discussion of Issues Arising from These Investigations

As mentioned in our introduction, from the experience of researchers in the field, including our own investigations reported in the literature review, several methodological issues have arisen during the evolution of interview design and technique, as follows:

- (a) The need for interviewers to be involved in any research design so that they are able to diverge from standard questions to explore concepts using Socratic dialogue. This demands in-depth, content knowledge of the field of observational astronomy (CK), as well as skill in interview technique exemplified by Piaget’s clinical method and proficiency in identifying opportunities for teaching (PCK) within Vygotsky’s ZPD. In harmony with Kvale and Brinkmann (2014), we believe that the interview as builder of knowledge through dialogue has been undervalued due to a number of factors such as lack of appropriate interviewing knowledge and skills, shortness of time and theoretical bias aimed at refuting the findings of others rather than making genuine contributions to human knowledge. The ‘proficiency’ of the interviewer (vis-à-vis the necessary CK and/or PCK) is however often unstated, many writers considering it unnecessary for that to be made clear. Whilst this is not to imply carelessness, it can be surmised from those few studies which do explicitly spell out the links between the interests of researchers and relevant school teaching, that it is a matter of concern. The relationships between interviewer CK/PCK and his/her skill in handling Socratic dialogue to best advantage merit close scrutiny (discussed further below).
  Implicitly in general terms, however, behind all of the investigations is the need to determine what children think in order to guide further teaching. Also implicitly, and of equal importance, is the desire on the part of educators to replace everyday ideas with scientific concepts, i.e. to bring about ‘conceptual change’ for the young people concerned. Sometimes the outcomes of research into children’s ideas are global in character with the design of a study seeking to inform curriculum change in a country or culture. At other times, the outcome is more local with reform taking place within a single school or class as part of a trial. Whatever the level of educational endeavour, design and application depend on teachers having high CK/PCK; and awareness of opportunities afforded within the ZPD.
- (b) The significant part played by *multimodal* methods and what can be interpreted from interviewees’ responses to in-depth questioning utilising *different* modalities. Since their introduction by Nussbaum and Novak (1976), children’s drawings have complemented the spoken word in many interviews. Similarly, the innovative use of clay modelling by Brewer, Herdrich and Vosniadou (1987) added a third modality from which to triangulate children’s ideas. Working independently, the current authors adopted a multi-modal methodology involving verbal language, children’s drawings and children’s play-dough modelling which has proved to be most successful (see text and protocols below).
- (c) How an understanding of the creative (as opposed to the reproductive) dimension of remembering—what recent neuroscience research tells us about the *dynamism* of human memory—alters our interpretations of what may be revealed when people are questioned. The main objective of interviews in science education research is to determine what children think to inform curriculum development and teaching strategies. A resultant aim is to replace children’s everyday ideas with scientific concepts—the aforementioned process of conceptual change. Bearing in mind that recent neuroscientific research has shown this to be somewhat mistaken since human memory does not forget old ideas but suppresses them in favour of more plausible ones to fit the immediate situation, everyday and scientific ideas co-existing in a *creative*, *dynamic* memory. What is required is to teach children how to discriminate between the two repertoires of ideas to make appropriate selections to fit specific situations. Interviews utilizing open-ended questions with Socratic dialogue reveal these co-existing concepts and provide the opportunity to guide children towards more scientific ways of thinking through scaffolding^4^ (with the interviewer in the role of teacher rather than impartial researcher^5^).
- (d) The consideration which should be given to the value of the spoken word as a reflection of conceptual ideas and the merits of open-ended over forced-choice questions. Whereas the child’s verbal responses to questions are of paramount importance to the Piagetian clinical method (see Piaget, 1926, 1929, 1930), and equate to thought in Vygotsky (1962), for some they do not enjoy such high status in radical socio-cultural methodology (see Schoultz et al., 2001). This contrast is exemplified by the different outcomes evident from open-ended questions generated from dialogue between interviewer and child utilised by Vosniadou et al. versus the closed or forced-choice interviews preferred by Schoultz et al. (the latter featuring the use of cultural artefacts such as globes or maps as props).

Addressing points (a)–(d) is important to science education because, notwithstanding great progress in neuroscience, the Piagetian interview remains the gold standard way of ascertaining what children think. Researchers need to be aware of the methodological difficulties encountered in interviewing and the guidance suggested by others active in the field in the past and present to ensure a successful outcome. In the sections which follow, we will now focus on these and other key issues which arise from the historical record. In doing so, we will illustrate the points with extracts of several protocols from interviews we have conducted. The text of each extract is left-addressed but the parts of the protocols which illustrate Socratic dialogue are indented (and marked ‘Start of …’ and ‘End of ….’). ‘*Main Discussion’* points relate to the main text of this article.

Interviewer knowledge and skill as teacher/researcher (CK & PCK): Scaffolding

Protocol Exemplar:

*Susana* (NZ Rotuman^6^: Female: Age 11 Years 8 Months): Sunrise & Sunset.

Questions about Observing the Sky during Daytime (NZ and Rotuma, Fiji): Outdoors.

*R. Have you seen the Sun rise?*

*C. Yes.*

*R. Where does the Sun rise?*

*C. I think it’s over there* (points to Tararua Mountains in the West).

Start Socratic Dialogue

*R. It rises over there* (I indicate West)*?*

*C. The West or something.*

*R. It rises in the West?*

*C. No—it rises in the South.*

*R. It rises in the South?*

*C. Because they say Dunedin* (a city on the South-East coast of NZ) *is the first place* *to get the new day.*

*R*. (I mention that I have heard similar claims made about Gisborne – a city on the NE coast of NZ – and I suggest that Susana does some research in class using her atlas to find the most Eastern point of NZ and hence the closest point to the International Date Line: in fact it is Gisborne but, in keeping with Socratic Dialogue, I don’t tell her).

*R. Where does the Sun set?*

*C. In the North.*

*R. Have you seen the Sun set?*

*C. Yes—on TV.*

*R. You haven’t seen the Sun set in real life?*

*C. Oh—in real life—I might have—back on the Island* (Rotuma—see Note below).

*R. Well—you can relate to the Island—have you seen the Sun rise on the Island?*

*C. Yes.*

*R. Have you seen the Sun set on the Island?*

*C. Yes.*

*R. But you don’t know—on the Island—whether it was East or West?*

C. *No*.

End Socratic Dialogue

### Protocol Discussion

The researcher’s knowledge of astronomy, local knowledge of geography and information from child’s teachers guides the Socratic dialogue.

### Main Discussion

The concept of teacher knowledge and skill has been of concern to teacher educators for over a century (see Bullough, 2001, for a review). Since its introduction by Shulman (1986, 1987), *Pedagogical Content Knowledge* (*PCK*) has been the subject of debate between teachers and educational researchers. Whilst ‘what to teach’ has been defined and supported by science curricula broadly described as Subject or *Content Knowledge* (*CK*); ‘how to teach’ (PCK) has been relatively overlooked for a variety of reasons not least because teacher knowledge and teaching skill are hard to define (see Abell, 2008; Abell et al., 2009; Barnett, 2003; Fernandez, 2014; and Neumann et al., 2019). In his criticisms of PCK from a historical perspective, Settlage (2013) wished that the concept had taken on a greater role operationally as subject matter knowledge *for teaching*; arguing that teachers need greater *contextualised* understandings of how students may be helped to learn. Addressing these concerns from the perspective of teacher/researchers, we believe that (whatever the constructs are called, ‘pedagogical’ being enigmatic) there is a need for teachers to not only know the essential knowledge of their teaching domain but also, a much more difficult task, have the ability to sense moments of readiness for learning and opportunities for scaffolding through Socratic dialogue. Subject knowledge is of limited value unless the teacher knows when it is relevant to the context; and how to teach it in a way that captures the imagination of students (see van Driel et al., 1998; Nilsson & Vikström, 2015; Neumann et al., 2019).

Focusing on the ideal prerequisites of researchers seeking to investigate children’s observational astronomy of the ESM, we are looking for teachers with a working knowledge of basic astronomy *and* an equally sound understanding of developmental psychology fit for educational purposes. The former is essential for the interviewer (as researcher) to devise knowledge probing questions for incorporation into Piagetian clinical interviews; the latter is needed to enable the interviewer (as teacher) to take advantage of children’s responses to teach by scaffolding within Vygotsky’s ZPD—either directly (breaking Socratic tradition) or indirectly by referring the child to his/her class teacher or librarian. This requires sensitive interviewing. Socratic dialogue can clarify children’s ideas for the interviewer, with the method enabling the child’s own construction of knowledge during the child-interviewer interaction. This principle is not easy to put into practice and it underlines the opening words of our argument that ‘an interview is an inter view’.

Considering the CK aspect either separately or as an integral part of PCK, the literature on research into children’s cosmologies considered above emphasizes the need for teachers to give children ‘direct experience with phenomena’ (Nussbaum & Novak, 1976, p. 549). Whilst the constraints of time and timetable structure limit the opportunities for children to carry out direct observational astronomy, the literature (including our own studies) do give some examples. For instance: (i) studying the divergence of the shadow of a shadow stick due to the apparent motion of the Sun as a result of the rotation of the Earth; (ii) observing the Moon in daytime against a fixed object such as a power pole or lamp post at the same time daily over a period of time to gain an impression of the motion of the Moon; (iii) recording the shape of the Moon over time, such as between interviews where these extend over more than a few days followed by classroom or library research of phases; (iv) viewing sunrise and sunset at home and (with parental co-operation) thinking in terms of Earth moving and with respect to the Sun rather than the Sun moving with respect to Earth; (v) noting the change in position of a constellation of stars over time (again with parental collaboration) and explaining the changes in terms of the rotation of the Earth; and (vi) observing Foucault pendulums: interesting phenomena demonstrating the rotation of the Earth which may be seen in some museums.

Multi-modal method utilising verbal language, drawing and play-dough modelling

*Protocol Exemplar:*

*Katherine* (NZ European: Female: Age 10 Years 8 Months): Shape of ESM.

Earth Shape Interview

*R. What shape is the Earth?*

*C. A circle.*

*R. Is it a circle like a ball, or a circle like a pancake?*

*C. A ball.*

Earth Shape Drawing

*R. Draw the Earth.*

**Fig. 1:**
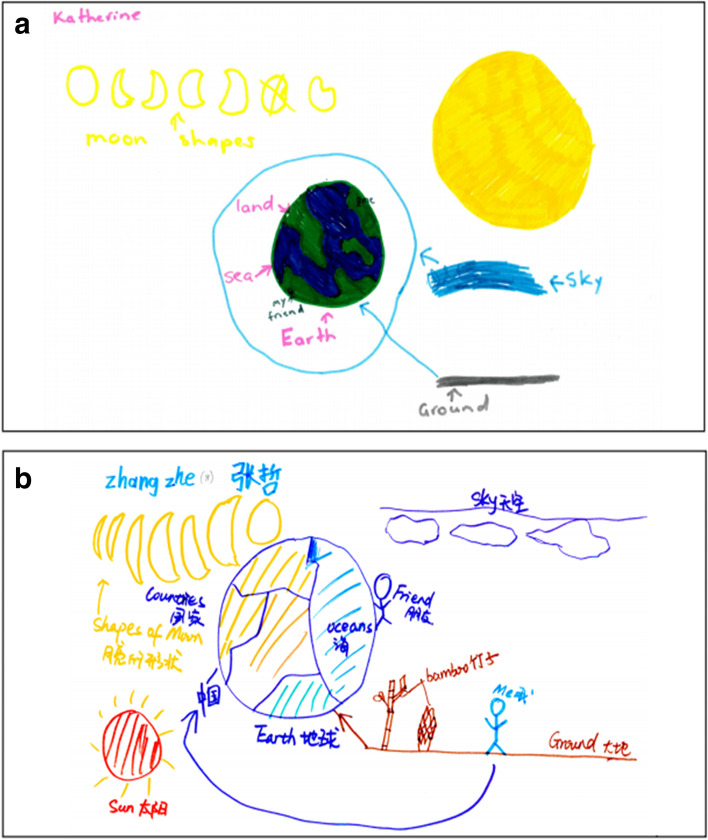
**a** Katherine (10 years 8 months). **b** Zhang Zhe (8 years 10 months)

C. (Draws) (see Fig. 1a).

Drawing Ground and Sky

*R. Could you draw the Ground and the Sky? *

C. (Draws ground horizontal below Earth and sky horizontal above ground adjacent to Earth).

*R. Where have you drawn the Ground?*

*C. On the green.*

Start Socratic Dialogue

*R. Why have you drawn the ground there?*

*C. Because the sky’s above the ground.*

*R. Is the Ground on the Earth?*

*C. Yes.*

*R. But you haven’t drawn it on the Earth, have you?*

*C. No.*

*R. Just draw an arrow indicating where it is up there.*

C. (Draws arrow from ground to Earth).

End Socratic Dialogue

R. Is the Sky part of the Earth?

C. Yes (draws sky around Earth with arrow from curved sky to circular sky).

### Note

Drawing Ground horizontally below Earth, and Sky horizontally above or below Earth are thought to be indicators of a flat-Earth cosmology (Nussbaum & Novak, 1976).

Earth Shape Modelling

*R. Could you make the shape of the Earth with the green play-dough?*

**Fig. 2:**
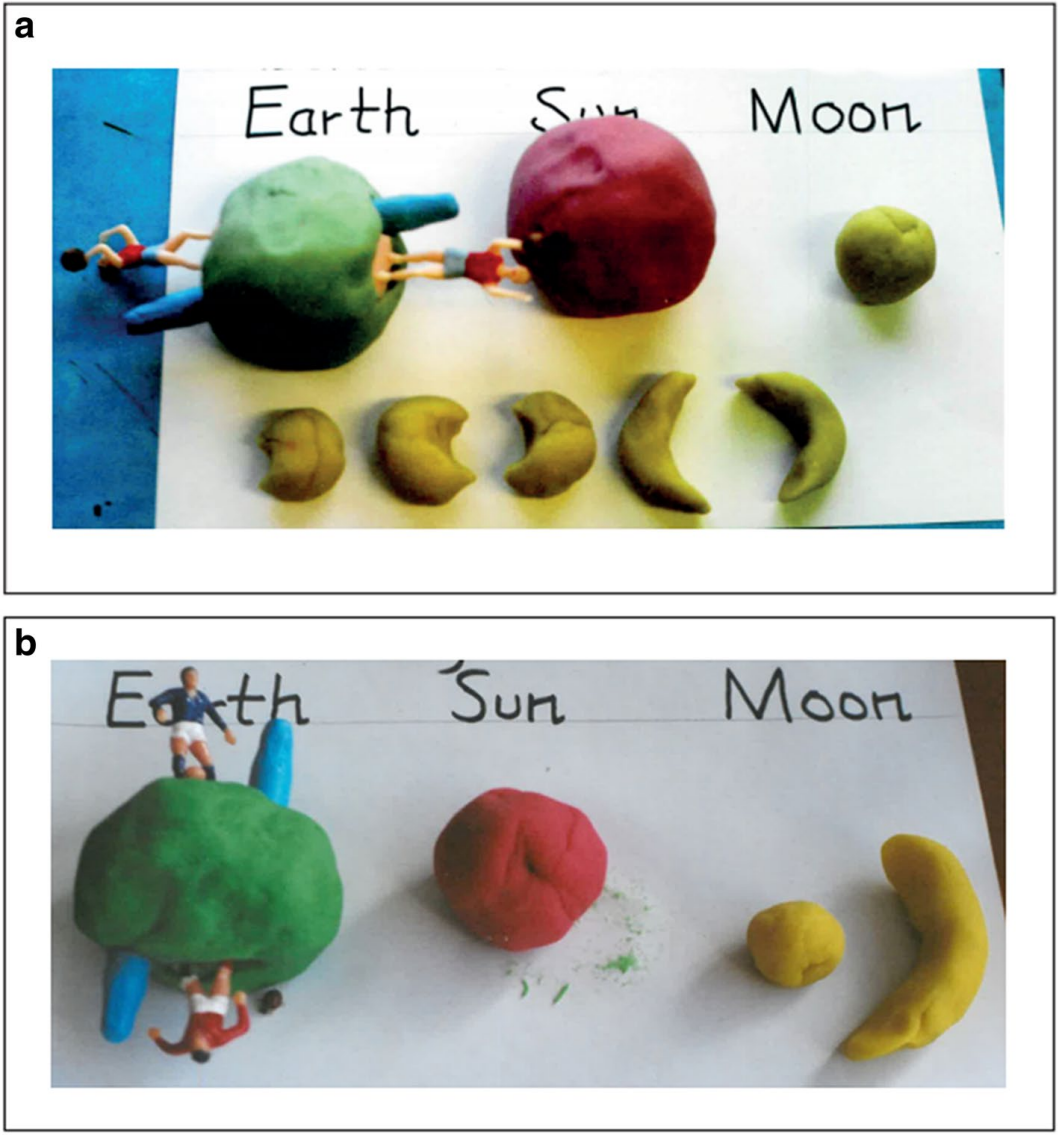
**a** Katherine (10 years 8 months). **b** Zhang Zhe (9 years 11 months)

C. (Models Earth ball-shaped) (see Fig. 2a).

### Main Discussion

Through dialogue, drawing and modelling the child develops their Earth concept with elements of physical shape, Ground and Sky, Habitation of Earth and Identity with Earth. By such multi-modal activities, any cognitive conflicts between concepts can be resolved to create a concept of the Earth for the current context. The retention of these multi-modal concepts is thought to be dependent on the neural pathways that created them through a process of re-entry (see Edelman, 2005, 2006). Access to the linguistic elements of the original pathways may depend on recognition of linguistic patterns in the questions (Cromer, 1987).

*Protocol Exemplar:*

*Zhang Zhe* (China: Han: Male: Age 8 Years 10 Months): Shape of ESM.

Earth Shape Interview

*R. Tell me about the Earth?*

*C. The Earth is shaped like a ball full of air – round.*

Earth Shape Drawing.

*R. Draw the Earth.*

C. (Draws) (see Fig. 1b).

*R. Draw the Ground and Sky.*

*C.* (Draws) (see Fig. 1b).

Earth Shape Modelling

*R. Make the shape of the Earth with the green play-dough.*

*C.* (Models Earth like a ball then flattens to a disc).

Start Socratic Dialogue

*R. Are you flattening it now?*

*C. Yes.*

*R. I thought it was like a ball?*

*C. The Earth is round like a ball but I thought I should shape it like my drawing.*

*R. Make it the shape that it really is.*

*C.* (Models Earth as a ball).

End Socratic Dialogue

Moon Shape Modelling

*R. Make the shape of the Moon with the yellow play-dough.*

*Now – with the Moon you’ve made (drawn) many shapes – if you could make two of them.*

*C.* (Models shapes of Moon – ball and crescent) (see Fig. 2b).

### Protocol Discussion

Zhang modelled the Earth as a ball, and then flattened it to a disc to match his drawing. Finally, through Socratic dialogue he decided that it was ball-shaped. This process suggests that he was comparing different concepts of Earth as he modelled. Further questioning on Identity with Earth confirms that he knows the Earth is spherical by his placing ‘Self’ and ‘Friend’: on opposite sides of the Earth; and indicating that gravity acts from the Earth’s centre.

### Main Discussion

Reasoning in multiple modalities involving comparison between concepts suggests rapid mental simulations (see Barsalou, 2003). Recalling concepts from a year before hints at processes such as Cromer’s (1987) linguistic pathways or Edelman’s (1987) re-entry theory to re-generate the concept. As described in the historical literature review, multi-modal methods have been used to investigate children’s cosmologies since the pioneering work of Nussbaum and Novak (1976) who utilised a globe, pre-made models, pictures and drawings of the Earth as props and asked children to draw the path of falling rocks and water from a spherical Earth. Following experience, they cautioned against the use of cultural artefacts which they found to inhibit children’s intuitive responses. Notwithstanding these reservations, a similar procedure was adopted by Nussbaum (1979), Nussbaum and Sharoni-Dagan (1981) and Sharp (1999).

Influenced by Nussbaum and Novak’s (1976) work, two groups of researchers independently developed multi-modal methods (incorporating verbal language, children’s drawings and children’s clay and play dough modelling) for investigating the emerging field of children’s cosmologies. These were Vosniadou and her colleagues working in Samoa, Greece, India and the USA (Brewer, Herdrich & Vosniadou, 1987; Vosniadou & Brewer, 1990, 1992, 1994; Samarapungavan et al., 1996; Diakidoy, Vosniadou & Hawkes, 1997); and the authors working in NZ and China (Bryce and Blown 2006). These media were also utilised by Skopeliti and Vosniadou (2007). Cultural artefacts in the form of globes and maps were central to the socio-cultural methodology used by Schoultz et al. (2001) and by Ivarsson, Schoultz and Säljö (2002) to critically question the methodology of Vosniadou et al. The results of using globes and maps as props appeared to confirm Nussbaum and Novak’s (1976) reservation that rather than illuminating children’s concepts cultural artefacts suppress intuitive concepts. Following their recommendations as far as possible, we avoided using cultural artefacts such as tennis balls as props unless children had already indicated that they believed the Earth to be spherical; and we avoided globes. The multi-modal methodology developed by the authors [exemplified by the protocols of verbal interactions of Katherine and Zhang Zhe, together with drawings (Fig. 1) and play-dough models (Fig. 2)], has proved to be particularly effective in probing children’s concepts of Earth shape and gravity as in Bryce and Blown (2013).

Creative remembering and dynamic memory

*Protocol Exemplar:*

*Brayden* (NZ Māori: Male: Age 9 Years 11 Months).

Questions about the Motion of ESM

*R. Is the Earth moving?*

*C. Yes.*

*R. How is the Earth moving?*

*C. I don’t know.*

Start Socratic Dialogue

Time Concepts

*R. Has a day got anything to do with the Earth and the Sun?*

*C. Yes.*

*R. What’s a day got to do with the Earth and the Sun?*

*C. The Earth turns around and the Sun doesn’t move so the world moves around the*

*Sun.*

*R. How long does the Earth take to turn around once?*

*C. A day.*

*R. What is a year?*

*C. It’s when the Earth goes round the Sun.*

End Socratic Dialogue

### Protocol Discussion

Although some of these questions in Socratic dialogue could be interpreted as ‘leading’, this must be weighed against their role in reminding the child of what they already know—as in the case of the rotation of the Earth which the child had ‘forgotten’.

*Protocol Exemplar:*

*Zhi Hong Yu* (China: Han: Male: Age 6 Years 11 Months): Viewing Earth from Moon.

Viewing the Earth from the Moon (China)

*R. Imagine that you were on the Moon and were looking at the Earth very carefully.*

*R. What could you see on the Earth from the Moon?*

*C. Part of it is blue and part of it is white.*

Start Socratic Dialogue

*R. Why?*

*C. I’m not sure.*

*R. Could you see Land or Continents?*

*C. No.*

*R. Could you see Oceans or Seas?*

*C. Yes.*

*R. Could you see Clouds?*

*C. Yes.*

*R. Could you see countries like China?*

*C. Yes.*

*R. Could you see large cities like Changchun?*

*C. Yes.*

*R. Could you see streets like Weixing Lu (Satellite Road)?* (site of Chang Da School).

*C. No.*

*R. Why not?*

*C. It’s too far away.*

End Socratic Dialogue

### Protocol Discussion

Zhi’s verbal responses suggest that he really imagines looking at the Earth from the perspective of the Moon changing focus dynamically and recalling detail from memory.

### Main Discussion

In our introduction, we posed the question of how understanding the creative (as opposed to the reproductive) dimension of remembering can alter our interpretation of interview responses (be they verbal language, drawings or play-dough modelling). In Bryce and Blown (2021), we drew attention to what recent neuroscience research tells us about the *dynamism* of human memory (see Footnote ^1^). We concluded that open-ended Piagetian clinical interviews conjoined with Socratic dialogue can yield insights into how children think, including the processes of imagery, memory and metacognition. These observations revealed that memory is in a constant state of flux with new ideas being compared with old in a competition for relevance to the interview situation and the question context. The evidence suggests that multiple repertoires of knowledge coexist in memory in two major domains: everyday, intuitive, cultural ideas and scientific concepts. The former earlier-learned notions are not replaced by the new ideas taught at school, rather they are inhibited as not appropriate (or the best match) to the context. We also found evidence of memory being non-representational as argued by Edelman, and concepts being akin to simulators or skills which operate consistently across several modalities (see Barsalou, 2003; Edelman, 1989, 2001, 2005). The dynamic nature of memory is evidenced by examples from children’s cosmologies where everyday concepts such as sunrise and sunset coexist in harmony with scientific concepts such as the rotation of the Earth: both describe the same phenomena but do so utilizing different perspectives and different language modes or repertoires. The classic case of radical knowledge restructuring put by Vosniadou and Brewer (1987) was ‘the change from a geocentric schema in which the earth is conceptualized as flat and motionless to a heliocentric schema in which the earth is conceptualized as spherical and rotating’ (p. 60). Whilst we found evidence of this and similar cases of restructuring (see Blown & Bryce, 2006), we also found that everyday concepts were not replaced but rather became less likely to be selected in a scientific context such as a Piagetian interview. Thus children’s memory has been found to be much more creative and dynamic than some previous studies suggested. Rather than change taking place gradually over extended periods of time (years), the evidence shows change occurring rapidly as children switch modes or repertoires more-or-less instantaneously in response to interview questions and Socratic dialogue see Blown and Bryce (2017). But to report these processes as ‘conceptual change’ would be a misnomer. The concepts have not changed: they have been re-prioritised in order of relevance to the context. A response is created by the child from all available information to generate what Edelman (2001) called ‘the remembered present’.

Value of spoken word in response to open-ended questions and Socratic dialogue

*Protocol Exemplar:*

*Daniel* (NZ European: Male: Age 10 Years 8 Months): Tidal locking of Earth-Moon.

Drawing ESM Motion

*R. Draw the Earth, Sun and Moon and if anything is moving show how it is moving.*

*C. (Draws).*

*R. Does the Moon move?*

*C. Yes.*

*R. How does the Moon move?*

*C. It moves round the Sun beside the Earth (unclear).*

*R. How long does it take for the Moon to go round the Earth once?*

*C. I think it’s one month.*

*R. Does the Moon spin?*

*C. Yes.*

*R. How long does it take for the Moon to spin once?*

*C. One month.*

Start Socratic Dialogue

*R. …if it’s spinning in a month and it’s going around the Earth in a month what does that mean about what we can see?*

*C. Yes?*

*R. Do you know whether we ever see both sides of the Moon – from the Earth?*

*C. I don’t know – I know that you can see a crescent from both sides – but…*

*R. Well – you look into the idea of whether you can actually see the other side of the Moon from the Earth - or whether we only ever see one side. So you might have to look in an atlas and have a look at photographs of the Moon.*

*C. Yes.*

*R. And see whether there are actually two sides shown.*

*C. Yes.*

*R. And then think again about what you’ve just told me- that the Moon spins in the same period as it goes around the Earth.*

*C. Yes.*

*R. Well think about that.*

*C. Yes.*

*R. If you want to come back to me you can.*

End Socratic Dialogue

### Protocol Discussion

During the interview, the opportunity arose to probe the child’s knowledge of the Earth-Moon relationship through Socratic dialogue; i.e. to explore whether the child might understand the concept of tidal locking of the Earth and Moon due to mutual gravity and the Moon’s period of rotation equalling its period of revolution (NASA, 2017).

*Protocol Exemplar:*

*Chen Zhuo* (China: Han: Female: 10 Years 8 Months): Sun size: researcher/teacher conflict.

Questions about the Shape, Nature, and Structure of the Sun (China).

*R. Tell me about the Sun?*

*C. The Sun is made of two elements—hydrogen and helium. The life of the Sun is 10 billion years. After 10 billion years the Sun will turn into a little white ball and another Sun will appear. The temperature inside the Sun is 6000*^*0*^*C—on the surface there are solar flares 50–60 km high.*

### Protocol Discussion

Chen mentioned radiant energy but is not familiar with nuclear fusion—although she knows that the Sun’s energy comes from hydrogen and helium.

Drawing the Sun (China)

*R. Draw the Sun.*

*C. (Draws the Sun).*

*R. How big is the Sun?*

*C. Very, very big.*

Start Socratic Dialogue

*R. Is the Sun bigger than the Earth?*

*C. Yes.*

*R. How many Earths would fit across the Sun?*

*C. Two.*

*R. (The teacher inside me wants to correct, because child is otherwise so*

*knowledgeable, but I continue my Socratic dialogue).*

*R. Why is the Sun hot?*

*C. There is a lot of hydrogen inside.*

End Socratic Dialogue

### Protocol Discussion

Like NZ children, most Chinese children were more familiar with the Sun than they were with the Earth. Chen demonstrated considerable scientific vocabulary in response to standard interview questions and Socratic dialogue.

### Main Discussion

In point (d) of our introduction, we reported that Schoultz et al. (2001) questioned the value of oral language. They did so from a radical socio-cultural perspective, one that seems to be out of kilter with Vygotsky who placed great value on both inner speech as thought and external speech as essential for the social construction of knowledge within the ZPD (Vygotsky, 1962). Traditionally, verbal language has been the mainstay of Piagetian one-to-one clinical interviews; and together with children’s drawings and models have been the standard method of sharing ideas about the world as described in children’s cosmologies. The spoken word has proved particularly helpful in clarifying complex ideas; or explaining cultural interpretations of phenomena; or capturing switching between cultural and scientific repertoires; particularly when responding to Socratic dialogue. The two aforementioned cases where verbal language needs support are when interviewing young children age 3–6 at kindergarten or pre-school; or when interviewing children in another culture using another language through interpreters. In these situations, a multi-modal methodology with drawing and modelling affords triangulation with verbal language (limited due to age, or subject to interpreter mediation with technical terms having to be simplified in real time during three-way Researcher-Interpreter-Child dialogue^7^).

A fourth modality employed with success by the current authors to complement the spoken word was video-recording of gesture particularly when modelling the shape and motion of ESM. Historically, gesture and language are strongly associated, and there is evidence that drawing and clay were used to share ideas as depicted in cave art and figurines. The evolution of hand and mind together is also manifest in tool making and was summed up by the philosopher A. N. Whitehead: ‘It is a moot point whether the human hand created the human brain, or the brain created the hand. Certainly the connection is intimate and reciprocal’ (cited by Donaldson, 1978, p. 83). Language is a central component of this development. Using everyday and scientific language precisely yet economically is one of the prerequisite skills demanded of researchers who design interviews. And this is accentuated when working in another culture with another language involving interpreters. Knowing which everyday term translates to which scientific word and vice versa requires the type of ability that Barsalou (2003) refers to when he associates concepts with skills. Sometimes words are inadequate as when (moving to the physical sciences) Heisenberg wrote to Bohr on the enigma of quantum theory: ‘we must realise that our words don’t fit’; and Bohr replied ‘words are all we have’ (Baggott., 2011, p. 101). Fortunately, the spoken word is usually able to meet the challenge of interpretation.

## In conclusion

The historical record of investigations into children’s cosmologies explored in this paper has revealed a rewarding vein of activity by researchers in science education. The work has contributed to how we understand young people think and develop intellectually as they wrestle with important scientific ideas that impinge on their daily lives (however ‘ordinary’ the concepts might be thought to be at a cursory glance). Also, and particularly through the more recent studies, it has revealed how complex and demanding interview strategies actually are. The methodological findings are widely applicable in our view and signal warnings to researchers and teachers about what we may take to be ‘understood’ when we listen to young people expressing their thoughts and answer our questions—whether they are speaking these thoughts, or drawing pictures to show how they see them, or making representative models of what is in their minds. The literature has certainly emphasised the importance of cross-referring between modalities as we search for ways to support children’s scientific learning. ‘Caution’ should be the watchword when we are tempted to conclude that *we now know* what a young person thinks on the basis of any short interchange we have shared, however focused we feel that exchange has been. Crucially, it is imperative that the interviewer has to be very well versed in the subject area concerned; capable of teaching it to children; skilled in distinguishing between when and when not to teach; and in particular skilled in using Socratic dialogue to give the interviewee opportunities to freely articulate his/her thinking—therefore proficient in manner and style to be seen as completely non-threatening in a setting conducive to friendly exchange.

The everyday repertoire of children’s ideas often encountered in interview situations stem from parents and grandparents in their own cultural contexts, as well as from class teachers, librarians and much of the media. Teacher/researchers however have a responsibility to contribute to the development of children’s scientific repertoires to appropriate levels, either by Socratic dialogue within the ZPD or by referring children to teachers and librarians for further knowledge. Teacher/researchers are also well placed to complement others in teaching children to distinguish between the two coexisting ways of interpreting and making sense of the world. Those (few) educational policies intent on replacing everyday ideas with scientific ones seem somewhat misguided in that they fail to recognise that active remembering and thinking accommodate *both* interpretations of nature. The challenge is to design interviews that afford children the opportunity to respond in either repertoire and for interviewers to encourage children to think multi-modally.^8^ If we are to respond to the challenge of Bruner (1960) that ‘any subject can be taught in an intellectually honest form to any child at any stage of development’ (p. 33), then researchers must be finely attuned to opportunities to scaffold scientific knowledge either directly or by referral to other scientific sources of learning (teachers and librarians).

From a discursive position (see Van Langenhove & Harré, 1999; Jones, 2012), one could ask: If interviews are inter views (or inter-views), what do children gain from the interview? Our own response is: ‘Clarifying their ideas through Socratic dialogue, children’s drawings and play-dough modelling’. Astronomy is a science that captures the imagination of children in a unique way, leading in some to a lifelong passion as professional and amateur astronomers. For them, pursuing the frontiers of knowledge such as the origin of the universe and space exploration (see Salimpour et al., 2021), the interview as inter view is a unique opportunity to kindle lifelong enthusiasm for science.

From the perspective of interviewer as researcher, the impact of the interview on learning is hoped to be minimal to enable children’s concepts to be ascertained in their purest form. Whereas from the view of interviewer as teacher, the interview is designed to elicit and clarify knowledge and, in some cases, become a teaching instrument utilising Socratic dialogue. Avoiding imparting knowledge is particularly relevant in longitudinal studies where conceptual change as a result of development, experience, teaching and learning is being ascertained by repeated measures—asking the same questions up to 5 years apart with survey and control groups. In the event, in our studies, we found that several participants found the interview and its associated activities to be a source of astronomy knowledge (see Blown & Bryce, 2020). And, despite efforts of impartiality, survey groups who were interviewed three times had more advanced concepts than controls who were interviewed twice; the enhancement being attributed to survey children being familiar with the interview context and terminology (see Blown & Bryce, 2010). Thus, although unintended, the interview as inter view proved to be a powerful teaching instrument supplementing cultural and classroom sources of knowledge with lasting effects. (see Footnotes 5 and 7 on conceptual enhancement as a result of repeated measures).

Finally, given the quantity and quality of work in the field to date, it might be reasonable to ask those science education researchers who are interested in children’s cosmologies if there is more to be done. Assuredly, *yes*, and for a variety of reasons. With respect to astronomical knowledge, new ideas and information continue to arise and surface in the mass media, many of which school pupils take notice of and which ‘intrude’ on what might be considered as conventional science topics. Recent highlights would include planetary moons, exoplanets, asteroids, black holes, gravitational waves, the accelerating, expanding universe, dark energy, dark matter and so on. That is to say, whilst they may strictly go beyond the syllabus, new phenomena and events bring overlapping material into debate, raise confusions and stimulate questions.

Furthermore, we tend to think conventionally about education—teachers individually managing lessons with classes of young people, with evermore sophisticated resources, simulation materials and computers at their disposal. However, *on*-*line learning* increasingly drives instruction with phone technology, ‘smart’ television, school intranet facilities and the Internet (home-accessed) changing the ways in which much science reaches young people, and shaping its very content. Also, what we now refer to as *social media* also influence what young people learn as science (and everything else besides). The shift is not simply dispositional in character, affecting interests and bias. What children are encouraged to understand and *believe* through sources other than their teachers is more than simply incidental or ‘other’ background material. The richness and vitality of what is encountered on a daily basis brings considerable challenges to teachers (for example, see Bryce and Gray, 2004 with regard to *biotechnological progress*; and Bryce & Day, 2014a, b with regard to *climate change*). Interviewers face new challenges in respect of what science is actually experienced by young people. Questions put to children need to be contextualised rather differently; the CK and PCK of future researchers will have to be very different from those of their predecessors. Additionally, and unexpectedly, the effects of the Covid-19 pandemic in 2020 and 2021 have massively interrupted traditional schooling. In the UK and elsewhere, teachers have had to create on-line teaching and work with young people through digital materials and email on an unprecedented scale. Part-time education has invaded lives dramatically, both at school and university, so-called blended learning being put in place in advanced countries like the UK. (Blended learning is the euphemism for the sub-standard arrangements which combine much-reduced, face-to-face instruction and on-line delivery.) The pertinent point here is that research interviewers have many new issues to consider in respect of the *sources* and *integrity* of what knowledge learners might possess. What pupils may, or may not, have learned in schools needs to be thought about differently from what pertained only a few years ago.

## Footnotes

1 See Brault Foisy et al. (2015), Fugelsang & Dunbar (2005), Dunbar, Fugelsang & Stein (2007), Potvin (2017), Shulman & Valcarcel (2012) on inhibition and coexistence; Dresp-Langley (2012) on non-conscious representations; Freeman & Skarda (1990a, b) on dynamicism versus representationalism; Hoenig et al. (2008) on conceptual flexibility and dynamic semantic maps; and Mareschal (2016) on neuroscience of conceptual learning in science and mathematics.

2 The historical literature was compiled from published papers, books and theses used as sources in our extensive study of children’s cosmologies. These were kept up to date by searching the internet using Google Chrome and academic databases such as ProQuest Central. A typical search of children’s cosmologies literature would include search items such as: children, cosmologies, science concepts, astronomy, Earth. Similarly a search of pedagogical literature would include: teachers, researchers, Content Knowledge (CK), Pedagogical Content Knowledge (PCK). A search of interview literature would include: interview methodology, Piagetian interviews, Socratic dialogue. And a search of cognitive development literature would feature keywords such as children, cognitive development, science concepts, Piaget, Vygotsky. In general peer reviewed papers were preferred but given that the studies cover a century or more of work this was not always possible. Hence publication dates were left open ended to ensure that the reviews were as inclusive as possible within the parameters defined by keywords. Implicitly, this was a review of empirical studies with the focus on the variety of interview techniques utilised within different research methodologies. These reflected an ongoing tension between two main theoretical groups: constructivism after Piaget and socio-culturalism after Vygotsky.

3 ZPD: referring to what the learner can do with the support of the teacher (or more accomplished peers); that knowledge or the skills being withdrawn as the individual achieves autonomy in that field.

4 Scaffolding: Foley (1994) traces the origin of the term *scaffolding* to the work of Vygotsky. It was popularised by Bruner (1985) who understood that, for learning to take place, suitable social interactional frameworks had to be provided—by adults/peers. Scaffolding is the assistance given to a child within the ZPD to bridge the gap between what the child already knows and what the child could know with adult help. ‘What the child is able to do in collaboration today he will be able to do independently tomorrow.’ (Vygotsky, 1987, p. 211).

5 Traditionally, interviewers (particularly teacher/researchers) avoided teaching even when conditions were ideal to do so. As reported by Bryce & Blown (2006), care has to be taken in longitudinal studies not to impart knowledge or change children’s ideas deliberately since that could confound the outcome of repeated measures (the use of survey and control groups might enable the influence of the interview as a source of knowledge to be assessed). Where possible, in response to children’s questions demanding scientific information, the researcher referred children to their class teacher or librarian. However, in cases where children were unlikely to be interviewed a second time (such as the control groups) the researcher might share information. Evidence from experience and the literature suggests that children see researchers as teachers whatever reservations teacher/researchers may have about teaching whilst interviewing. We found that despite avoiding deliberate scaffolding, our interviews did influence the outcomes in longitudinal studies; the survey groups who were interviewed twice having more advanced concepts than the control groups who were interviewed only once.

6 Rotuma is an island dependency of Fiji, situated in the South Pacific Ocean about 440 miles north–north-west of Suva, the Fijian capital. The main island, Rotuma, which is volcanic, is 17 square miles in area, and is served by a small port. The island has a colourful history: in 1791 it was sighted by the lookouts of the *Pandora* whilst searching for the *Bounty* and her mutineers. The islands were annexed by Fiji, then under British control, in 1881. However the islanders retain their own cultural identity, hence Susana’s wish to be called Rotuman rather than Fijian (J. Mc Donald, personal communications, 5 & 12 August, 2001; Murray, 2008).

7 Significantly, although there have been several cross-cultural studies, and some longitudinal studies, only those by the current authors have employed both an ethnographical approach involving relatively long periods being spent by one of the authors as a teacher and researcher in both cultures; and longitudinal repeated measures requiring tracing of participants and follow-up interviews. Although intended to illuminate and measure conceptual change over time purely as a result of teaching and development, with the interview and interviewer of neutral influence (as objective researcher rather than interactive teacher); as mentioned above we found that in fact repeated measures resulted in enhanced conceptual knowledge in survey groups (interviewed twice) over control groups (interviewed once). This result highlights an area of particular interest to us, namely changes taking place within individuals as a result of interview questions or Socratic dialogue which are part-and-parcel of qualitative research investigations.

8 Although, in general we designed and conducted our interviews in scientific mode we made use of cultural stories, legends and folklore to encourage children to share ideas (such as the story of Alice in Wonderland by Lewis Carroll to probe gravity concepts; the Māori legend of Maui capturing the Sun to illuminate concepts of the Sun; and folklore on Chinese Festivals to explain phases of the Moon). Notwithstanding the reservations of some researchers to put aside their objectivity, the reality is that young children see researchers as teachers (as many are). Fortunately, techniques such as Socratic dialogue permit the researcher some flexibility to teach without compromising tradition.
